# Correlation Study between Multi-Scale Structure and *In Vitro* Digestibility of Starch Modified by Temperature Difference

**DOI:** 10.3390/foods13132047

**Published:** 2024-06-27

**Authors:** Yongting Feng, Meijuan Xu, Dongwei Chen, Xiao Zhang, Bin Zhou, Jian Zou

**Affiliations:** 1 College of Food and Biological Engineering, Henan University of Animal Husbandry and Economy, Zhengzhou 450046, China; foodilike@163.com (Y.F.); xumeijuan113@163.com (M.X.); 2 School of Life and Health Sciences, Hubei University of Technology, Wuhan 430068, China; cdw1773940897@163.com (D.C.); zhoubin4111@163.com (B.Z.); 3 Henan Heshenghe Food Co., Ltd., Xinxiang 453500, China; chuang861119@163.com

**Keywords:** modification, amylopectin, amylose, resistant starch, morphology

## Abstract

Physical techniques are widely applied in the food industry due to their positive impact on food quality and the environment. Temperature differences can effectively modify starch, but the resulting changes in starch structure and quality remain unclear. In this study, the corn starch was processed with high temperature, low temperature, and temperature difference (TD), including high temperature before low temperature (H-L) and low temperature before high temperature (L-H). The results showed that high temperature induced the umbilicus to concave inward shape and sharply decreased the amylose content, while low temperature increased the surface micropores and reduced the A-chain. TD reduced the fluorescence intensity and increased the clearness of the growth ring. TD elevated the relative crystallinity (RC), short-range order, A/B_1_ chains, hydrolysis parameters, and resistant starch (RS), and reduced amylose content, B_2_/B_3_ chains, and viscosity. Moreover, the corn starches treated by H-L had lower amylose content and higher RC, 1047/1022, A-chain, and RS than those treated by L-H. Overall, high temperature degraded the amylose and low temperature destroyed the amylopectin. During the TD, H-L can accelerate the starch molecular rearrangement more than the opposite temperature treatment order. These results will help produce novel starches for better food applications.

## 1. Introduction

Starch has some deficiencies when applied in the food industry, including easy regeneration and less stability at hot and cold temperatures. To avoid these defects, physical modification, chemical modification, and enzyme modification have been suggested. In recent years, there has been a lot of interest in clean-label food products [1]. Therefore, physical modification, as a safe processing technique, has become a major area of concern, and starch with physical modification can be considered a clean-label food. Freezing, as a preservation technique, is commonly used in starch-based foods, such as noodles. In addition, freezing is a desirable physical processing technique for modifying starch due to its mild and environmentally friendly process conditions [2]. As a pretreatment technique, freezing can not only slow down most biochemical reactions and reduce nutrient loss, but it can also ensure that food remains of good quality for consumption in the supply chain [3]. Freezing modification disrupts amorphous regions, leading to molecular rearrangement and thereafter improving short-range ordering [4]. The structure properties of food components will be impacted by ice crystal growth and water immigration in storage at −34 °C, thus influencing the food quality [5]. Ice crystals formed during freezing by liquid nitrogen can squeeze the starch particles, causing amylopectin to leak onto the outer layer of the starch particle, thereby improving the water absorption capacity of starch [6]. Furthermore, freeze–thaw cycles have been found to improve the resistance of ginger starch [7]. Additionally, a study revealed that compared to rolled oats frozen at −20 °C and −40 °C, rolled oats chilled at −80 °C had the best quality after steaming [8]. Hence, the alteration of the starch granule structure triggered by freezing treatment will influence the quality of starch-based food.

In addition, the effects of thermal processing on starch and food are of concern. The temperature and duration of high-temperature baking (80–230 °C) and deep-frying (140–200 °C) can affect the color, structure, and composition of the dough to varying degrees [9]. Researchers were also concerned about the stability of the oxidative quality of fried peanut oil up to 230 °C and the risk of chronic diseases [10]. Additionally, the heat-moisture treatment is a high-temperature (84–120 °C) and low-humidity (below 35%) thermal modification technique [11], which increases the fluidity and helical structure of starch chains, resulting in variations between crystalline and amorphous fields of starch particles [12]. However, when starch-based foods are treated at high temperatures, such as 230 °C, the change in the properties of starch is not known.

For the effect of high- and low-temperature difference on starch properties, it has been shown that the autoclaving–cooling technique favors the production of resistant starch [13]. However, for pregelatinized starch, freezing treatments (−20 °C, −80 °C, and −196 °C) were performed, and it was found that liquid nitrogen freezing improved the water-summing capacity of the starch but increased the GI value compared to other freezing temperatures [14]. In addition, it was discovered that two freeze–thaw cycles of pre-fermented frozen raw dough resulted in increased digestibility [15]. However, the effect of a larger range of temperature treatments and the order of temperature treatments on starch properties is worth investigating.

Quick-frozen prepared foods, such as small crispy meat and fried chicken, have been widely loved by young consumers [16,17]. Starch, the main component of the surface of coated food, undergoes low-temperature and/or high-temperature processing technology during processing. The above-mentioned studies have shown that the high-temperature upper-limit treatment temperature of 230 °C and low temperature of −80 °C affect starch-based food products, respectively. Therefore, this study aims to explore the multi-scale structure and digestibility changes of starch after temperature difference treatment, which will provide theoretical support for developing the quick-frozen food industry.

## 2. Materials and Methods

### 2.1. Chemicals and Samples

Corn starch with a purity of 98.78% was purchased from Hengrui Starch Co., Ltd. (Luohe, China). The α-amylase (from porcine pancreas) was obtained from Shanghai Yuanye Bio-Technology Co., Ltd. (Shanghai, China), 8-aminopyrene-1,3,6-trisulfonic acid (APTS) was from Sigma Aldrich (St. Louis, MO, USA), and amyloglucosidase was collected from Shanghai Macklin Biochemical Technology Co., Ltd. (Shanghai, China). The D-glucose assay kit (GOPOD-Format) was from Megazyme Co., Ltd. (Bray, Ireland).

### 2.2. Preparation of Samples

Corn starch was mixed with 20% distilled water in a tin can and left to equilibrate at 25 °C for 24 h. The pretreated corn starch was modified using the following methods (Table 1).

### 2.3. Morphology

#### 2.3.1. Micromorphology Observation

The surface structures and granule sizes were scanned by a scanning electron microscopy, as stated in the previous research [18]. Samples adhered to the sample carrier were sprayed with gold and then visualized by SEM. The scans of starch granules were captured at two magnifications (1000× and 3000×).

#### 2.3.2. Confocal Laser Scanning Microscopy

Samples were dyed with sodium cyanoborohydride (1 M, 4 μL) and 8-aminopyrene-1,3,6-trisulfonic acid, trisodium salt (APTS, 10 mM, 4 μL). The dyeing process lasted 18 h at 30 °C in a water bath. After the end of dyeing, the samples were washed with distilled water. Finally, the samples were suspended in a glycerol–water mixture (1:1) before scanning.

### 2.4. Chain Length Distribution (CLD) of Amylopectin

Chain length distribution of amylopectin referred to the previous study, with slight modifications [19]. In a boiling water bath, the 2.5 mL starch suspension (1 mg/mL) was gelatinized for 1 h. The starch paste was allowed to cool to room temperature before being mixed with sodium acetate buffer (125 μL, 400 mM, pH 5.4), Amossa isoamylase from starch pseudomonas (5 μL, 10,000 U/μL), and 10% *w*/*v* of sodium azide. The mixture was cultured at room temperature (25 °C) for one day with 10% *w/v* sodium borohydride after being equilibrated for 24 h at 38 °C. After drying this solution, the substance was dissolved in 200 μL of NaOH (0.1 M), which was performed using HPAEC-PAD.

### 2.5. Crystalline Structure

#### 2.5.1. X-ray Diffraction (XRD)

The diffractometer was used to scan the samples over a range of 4–60° (2θ) at a speed of 6 °/min. The device was operated at 30 mA and 40 kV. The software Jade 6 was utilized to determine the samples’ relative crystallinity.

#### 2.5.2. Fourier Transform Infrared Spectroscopy (FTIR)

The FTIR spectra were scanned separately for each sample. The wavelengths ranged from 400 cm^−1^ to 4000 cm^−1^ and the spectra were obtained after 64 scans at a resolution of 4 cm^−1^. The samples and KBr were dried for 12 h in an incubator at 45 °C. Then, each sample, mixed with KBr at a ratio of 1:200, was prepared in a tablet press and then spectroscopically tested by FTIR.

### 2.6. Amylose Content

Based on previous research [20], 10 mg of the sample was combined with 900 μL of sodium hydroxide solution and 100 μL of alcohol. The mixture was then cooked in water while being agitated for 10 min. After chilling, the volume was adjusted to 10 mL. Acetic acid (0.1 mL) and potassium iodide (0.2 mL) were added after the supernatant (0.5 mL) was extracted. After bringing the mixture to a steady 10 mL, it was allowed to sit at room temperature for 10 min. The sample’s absorbance at 720 nm was tested using a UV spectrophotometer.

### 2.7. Pasting Properties

To summarize, 3 g of the dry-weight sample was mixed with deionized water to reach a total dosage of 28 g using the RVA instrument (RVA4500, Shanghai Riphane International Trade Co., Shanghai, China). The specific testing procedures for the starch paste were based on the previous protocol [18]. Data were obtained through TCW3 software (Thermocline for Windows11.2, Newport Scientific Pty. Ltd., Warriewood, NSW, Australia) developed by Platinum Elmo Enterprise Management Co., Ltd. in Shanghai, China.

### 2.8. In Vitro Digestibility

#### 2.8.1. Starch-Type Detection Analysis

The starch suspensions, consisting of starch samples (50 mg) and phosphate buffer (10 mL, 0.5 M, pH 5.2), were stirred at 37 °C for 10 min. α-Amylase (4 mL, 3000 U/mL) and saccharifying enzyme (1 mL, 2500 U/mL) were added to the above mixture for enzymatic hydrolysis at 37 °C. Then, 0.5 mL of enzymatic hydrolysate was taken out at different time points (10, 20, 30, 60, 90, 120, 150, or 180 min). The samples from each time point were added to the centrifuge tube with 0.5 mL of sodium carbonate solution (0.3 M, 0.5 mL). Finally, the centrifuge tubes were centrifuged at 1000 g for 10 min. The supernatant (0.1 mL) and 30 times the volume of GOPOD solution were reacted in a 40–50 °C water bath for 20 min. Then, the absorbance value of the sample was obtained through an enzyme-linked immunosorbent assay (ELISA) reader, and the glucose content was calculated [21]. The glucose content of the sample at 20 and 120 min was used to calculate the RDS, SDS, and RS content, using the following formula:

(1) $$\mathrm{RDS}\left(\%\right)=\frac{G_{20}-M}{T}\times 0.9\times 100$$

(2) $$\mathrm{SDS}\left(\%\right)=\frac{G_{120}-G_{20}}{T}\times 0.9\times 100$$

(3) $$\mathrm{RS}\left(\%\right)=100-\mathrm{RDS}-\mathrm{SDS}$$

RDS: the starch was hydrolyzed in the first 20 min; SDS: the starch was hydrolyzed in the first 120 min; RS: the starch could not be digested in the first 120 min; M: free glucose content in starch before hydrolysis; T: total starch content of samples; G_20_ and G_120_: glucose content at 20 min and 120 min.

#### 2.8.2. Fitting to First-Order Kinetics

Referring to the protocol in [19], the digestion hydrolysis process of samples can be further emphasized by the first-order kinetic equation: [C_t_ = C_∞_ (1 − e^−kt^)], and logarithm of slope (LOS): [ln(dc/dt) = −kt + ln (C_∞_k)], where C_t_ is the proportion of starch digested at any time, C_∞_ presents the evaluation peak value of starch digested at the termination of the reaction, and k is the digestion rate factor.

### 2.9. Data Analyses

Minitab 19 was used to conduct a significance analysis. The charts were captured with Origin 2023b and Microsoft Office PowerPoint. The results were displayed as means ± SD. Data were duplicated at least three times. Nonlinear least square (NLLS) is the most common method used to estimate kinetic parameters [22]. Principal component analysis (PCA) was used to analyze the correlation between the indicators by Origin 2023b.

## 3. Results and Discussion

### 3.1. Morphology

**Figure 1:**
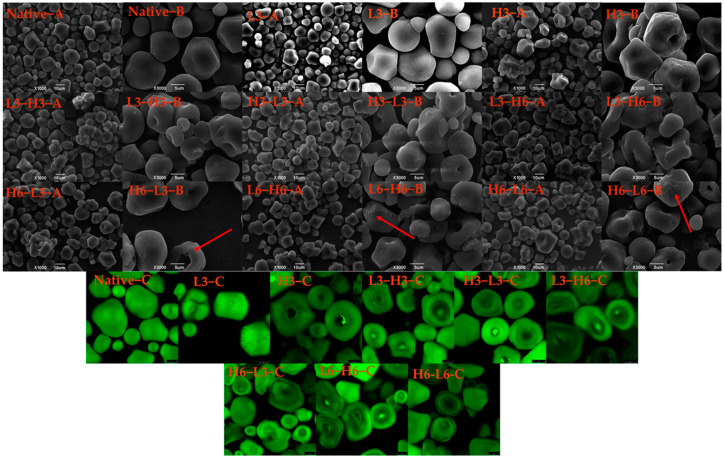
SEM (A,B) and CLSM (C) images of native and modified corn starch. The red arrows represent the appearance of micropores on the particle surface.

The morphologies of native corn starch, H3, L3, and TD starches, are displayed in Figure 1 A,B. The native corn starch presented a multilateral shape and smooth surface, which was consistent with the previous result [23]. When subjected to low temperature for 3 h, the granules of the starch remained unchanged, while H3 granules appeared to aggregate, and the hilum recessed inwards. In contrast, rice starch and red adzuki bean starch treated at 110 and 120 °C showed no signs of umbilical concave, only rupture, surface scallop, or agglomeration [11,24]. The central hilum of starch is primarily composed of amylose, and the central hilum consists of the amorphous region of starch, which is relatively fragile, especially in heating [25]. Therefore, high temperature led to the destruction of the amorphous region, rather than low temperature.

During the dual-modification process, as the low-temperature time (from L3-H6 to L6-H6, and H6-L3 to H6-L6) increased, the micropores (red arrow) were found in the outer layer of corn starch (L6-H6 and H6-L6). Additionally, with the extension of the high-temperature time (H3-L3 to H6-L3), the hilum severely recessed inwards, and the cavity grew. Therefore, the duration of dual-modification treatment could cause varying degrees of amylose damage, which was also confirmed in subsequent amylose analysis (Section 3.6). During the dual-modification, the processing sequence of high temperature and low temperature generated different effects on the surface granules. The surface characteristics and inner pore structure of the granules significantly affect the enzymatic efficiency and enzymatic hydrolysis formula of starch [26]. Therefore, it is clear that the different surface structures of starches resulting from temperature variations would significantly impact their digestibility.

### 3.2. Microstructure

In Figure 1C, the internal structure of starch granules can be analyzed, such as hilum, growth rings, amylose and amylopectin distribution, and reduced ends’ localization [27]. Generally, amylose reacts more with APTS and exhibits a stronger fluorescence intensity compared to amylopectin. The fluorescence intensity in the hilum region was higher than that in other regions due to a higher proportion of amylose [28]. The hilum of the H3 sample had a weaker fluorescence intensity, and the fluorescence intensity of L3 granules was relatively uniform, indicating the amylose of the hilum region was more sensitive to heat. The study pointed out that the hilum was susceptible to environmental influences due to its high water content and softness [29]. Besides, TD reduced the fluorescence intensity of corn starch. The decreased fluorescence intensity contributed to the rearrangement of molecular chains and fewer reducing ends [30].

The alternating light and dark growth rings did not appear in the native corn starch. The growth ring structure was related to the starch’s origin [30]. The growth rings surrounded by the hilum were found in the corn starches treated by H3 and TD, signifying the alternating arrangement of the amorphous and crystalline regions [31]. TD increased the visibility of the growth ring, showing that dual-modification could impact the internal structure and facilitate the rearrangement of amylose and amylopectin. Particularly for starches of H6-L6 and L6-H6, a more prominent growth ring was evident. This occurrence was largely dependent on the duration of TD, with longer durations having a greater impact on the crystalline and amorphous regions. Additionally, compared with L-H starches, the H-L starches exhibited lower fluorescence intensity in the umbilical cord, resulting from the break of amylose after heating treatment, which was also confirmed by the structural changes observed in the granular hilum through SEM. The related research found that the dry heat treatment reduced the distribution of reducing ends of amylose and amylopectin at the center of starch granules, as well as the double-helix’s fluidity [32].

### 3.3. Amylopectin Chain Length Distribution (CLD)

Table 2 summarizes the CLD of native corn starch and modified samples. The CLD included short A chain (DP6-12), medium-long chain B_1_ (DP13-24), B_2_ (DP25-36), and long-chain B_3_ (DP > 36). The double-helix of monoclinic cells from an A-type starch is mainly composed of A and B_1_ chains, while the double-helix of hexagonal cells from a B-type starch is mainly composed of B_2_ and B_3_ chains [19,30]. High temperature could not change the CLD, and low temperature decreased the A chain. It also suggested that low temperature damaged the short A chain of amylopectin rather than high temperature. The increased short chains could also improve starch’s resistance to digestion [33]. In the case of TD, an interesting phenomenon occurred, where the A and B_1_ chains increased while the quantity of B_2_ and B_3_ decreased. The increased A and B_1_ chains helped form double-helixes to increase the crystalline structure’s stability.

The A, B_1_, and A + B_1_ chain contents of the H-L samples were higher than those of the L-H samples, and the B_2_ + B_3_ chain contents of the H-L samples were less than those of the opposite treatment samples. The increase in short chains or the decrease in long chains could be obtained by starch crystallization [34], which was proven by the RC in Table 3. Besides, this illustrates that both H-L and L-H could facilitate the rearrangement of starch chains, but the degree of arrangement was higher for H-L than L-H. As the processing time prolonged, the proportion of A + B_1_ in TD starch increased, and the proportion of B_2_/B_3_ decreased, indicating that long chains were more susceptible to the influence of TD and degraded into A and B_1_ chains.

### 3.4. Crystallinity

**Figure 2:**
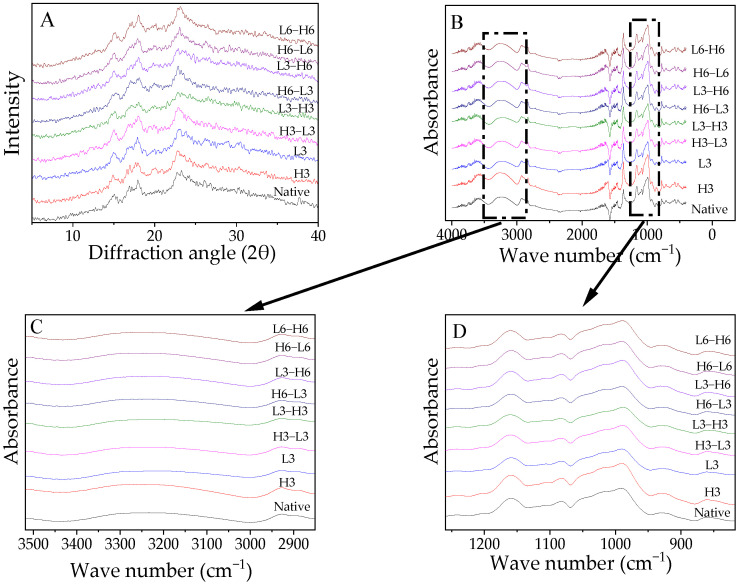
X-ray diffraction patterns (**A**), FTIR spectra (**B**), and deconvolution graph (**C**,**D**) of FTIR spectra from native and modified corn starch.

The XRD diffractograms are presented in Figure 2A and the RC is displayed in Table 3. Native corn starch exhibited a characteristic A-type XRD pattern, which was consistent with the conclusion of Chen et al. [35]. Generally, temperature modification did not affect the diffraction structure of starch because the collapse of the starch crystal structure requires sufficient water and gelatinization temperatures.

The RC of samples treated with L3 decreased, which was similar to the result from potato starch [6]. This indicated that ice crystals formed during low-temperature treatment impaired the A chain (Table 2), leading to decreased crystallinity. Studies have shown that a low temperature for 90 min could reduce the damage to granules [8], and the opposite result might be due to the duration. H3 could not change the RC. This suggested that although the temperature of the high-temperature treatment was very high, it still could not cause the gelatinization and degradation of starch due to the lack of water. The result was also in line with the CLD in Table 2.

Especially, H-L induced a higher RC than L-H. The short-chain fragments increased, encouraging interaction between the chains of starch to create a new, densely structured structure that increased stability against digestion [36]. Compared to the L-H samples, in the H-L treatment, the initial high-temperature treatment could not change the level of the A chain and maintained a higher content than the low-temperature temperature, which offered additional opportunities for rearrangement to form more double-helixes due to the temperature variance caused by the subsequent low-temperature treatment. Moreover, a prolonged treatment time formed more crystallinity, which resulted from the increased rearrangement effect, which was consistent with the results in the A-chain section mentioned in Section 3.3.

### 3.5. FTIR Spectra

The FTIR spectra of the samples are demonstrated in Figure 2B–D. The spectra of the treated starch did not exhibit significant changes regardless of processing time or sequence (Figure 2B). The absorption bands at 1022 cm^−1^ and 1047 cm^−1^ represent -CH_2_ bending and C-O-H vibration, indicating sensitivity to amorphous and crystalline structures [37]. The molecular arrangement of the starch at the short range could be represented by 1047/1022. The ratio value of H3 was higher than that of native corn starch, which might verify these findings by increasing RC (Table 3). From the deconvolution graph in Figure 2C,D, the absorption peak intensity of the modified starch changed to varying degrees around 3100–3700 and 995 cm^−1^, suggesting that the flexural oscillation of -OH caused a change in the hydration level in the crystalline zone [38].

High temperature could not significantly impact the 1047/1022, but low temperature reduced the 1047/1022, indicating that ice crystals during low-temperature treatment interfered with the order of the starch. TD raised the ratio of 1047/1022. Regardless of the order of treatment, an extended heating or freezing time improved the short-range ordering of the molecules. Additionally, the 1047/1022 of H-L was significantly higher than that of L-H, especially for the samples treated for 3 h, which demonstrated that H-L contributed to the development of more double-helixes [39].

### 3.6. Amylose Content

The content of amylose in the sample is shown in Table 3. The amylose content decreased for all modified starches. Among them, the L3 treatment had no significant effect on the amylose content in corn starch, while the H3 treatment significantly reduced the amylose content. The amorphous region was made up of amylose chains and branching amylopectin segments [40]. This suggested that thermal energy damaged amylose preferentially, which destroyed the amorphous region.

After TD treatment, the amylose content in H-L was noticeably lower than that of L-H. The starch-treated low-temperature treatment created higher amylose content. This increased the contact opportunities of amylose and amylose/amylopectin and allowed the amylose and amylose/amylopectin to efficiently rearrange, which could form a more stable and perfect crystalline structure. In addition, amylose avoided digestion in the body, so higher levels of amylose contributed to the anti-digestive properties of starch [41]. Some studies have found a negative correlation between amylose and crystallinity [40]. The RC results of this study were consistent with this trend. Besides, CLSM showed that the fluorescence intensity of L-H starch was higher than that of H-L, which was positively correlated with the conclusion of amylose.

### 3.7. Viscosity

According to Table 4, the peak viscosity (PV) of the sample treated with L3 showed no change compared with native corn starch. The PV reduced sharply in the presence of H3. During the heating process, the high temperature disrupted the amorphous region, making the remaining crystalline region difficult to absorb water and expand, and the final viscosity was much lower. Moreover, the PVs of H-L samples were higher than L-H ones. This may be due to the appearance of micropores on the surface of the L-H starch granules causing a decrease in viscosity [42] (Section 3.1). The heating and freezing cycles could also contribute to the formation of crystalline regions. These findings were consistent with CLD, RC, and 1047/1022 (Section 3.3, Section 3.4 and Section 3.5).

Except for the L3 sample, there were differences in the setback (SB) of all modified starch compared to the native starch. The SB is important, as it indicates the potential retrogradation of starches. The stability of starch paste is generally demonstrated by its breakdown (BD) value. The BD and SB values of corn starch induced by L3 were not markedly distinct in comparison to the native starch. This meant that the disintegration degree in terms of water absorption, expansion, and the retrogradation trend of L3 particles was similar to those of the native starch. Additionally, the gelatinization temperatures (GT) of H3, H3-L3, and L3-H3 were higher than that of the native starch, indicating that the modifications made it difficult for the starch granules to swell after absorbing water [19]. This phenomenon was due to the emergence of dense crystalline structures. However, the 3 h thermal decomposition cycle led to no significant differences in the GT values. Surprisingly, the GT value of the 6 h modified starch was undetectable. This might be due to the internal structure of starch particles being difficult to decompose, leading to recrystallization, which improved the stability of starch gelatinization [25].

### 3.8. RDS, SDS, and RS

**Figure 3:**
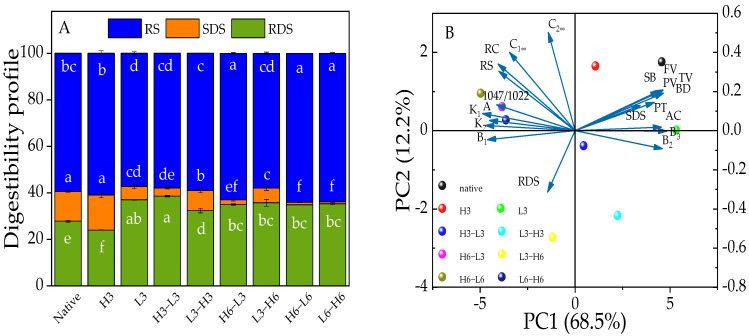
The digestibility profile (**A**) and PCA outputs (**B**) of native and modified corn starch. Different lowercase letters (a–f) represent significant differences in RS, SDS, and RDS among samples.

Starch can be categorized into RDS, SDS, and RS based on how long it takes for enzymes to digest them [43]. SDS intake can cause the increase of blood glucose more slowly than RDS, and RS can achieve the purpose of effective control of blood glucose [44]. The RS fraction is primarily composed of a well-structured crystalline form. The SDS component is made up of both amorphous and crystalline structures, while the RDS structure mainly consists of amorphous and dispersed starch [45]. H3 showed a trend of converting RDS into SDS and RS, and L3 could turn SDS and RS into RDS, while TD processing could convert SDS into RDS and RS (Figure 3A). Additionally, the results from Section 3.4 indicated the devastation of L3’s crystalline region and an increase in amorphous areas, resulting in reduced RS and increased RDS. A heat-moisture treatment at 120 °C, with a moisture content of 30% for 2 h, reduced the RS content and increased the RDS content of red adzuki bean starch [11]. However, the heat-moisture treatment can convert RDS into SDS and RS to slow down the digestion of corn starch [36]. Noteworthy, although the TD did not produce more SDS, it did convert more RS, demonstrating that the TD could produce a more well-structured form, which was demonstrated by the relative crystallinity following the same trend in this study.

Compared with TD samples, it was found that the RS contents of H6-L3/6 samples were higher than L3/6-H6 (Figure 3A), and the RS content of H3-L3 or L3-H3 samples was reduced. It was explained that 3 h of TD can damage the crystalline structure of RS, while 6 h of TD may cause the starch to change from a disordered to an ordered structure, producing more RS [46]. Therefore, the duration of the TD processing was an important factor in determining the resistance to starch digestion. Furthermore, it was speculated that once the starch structure was frozen after heat treatment, freezing shrinkage might further enhance the rearrangement of the starch.

### 3.9. First-Order Kinetics Analysis

**Figure 4:**
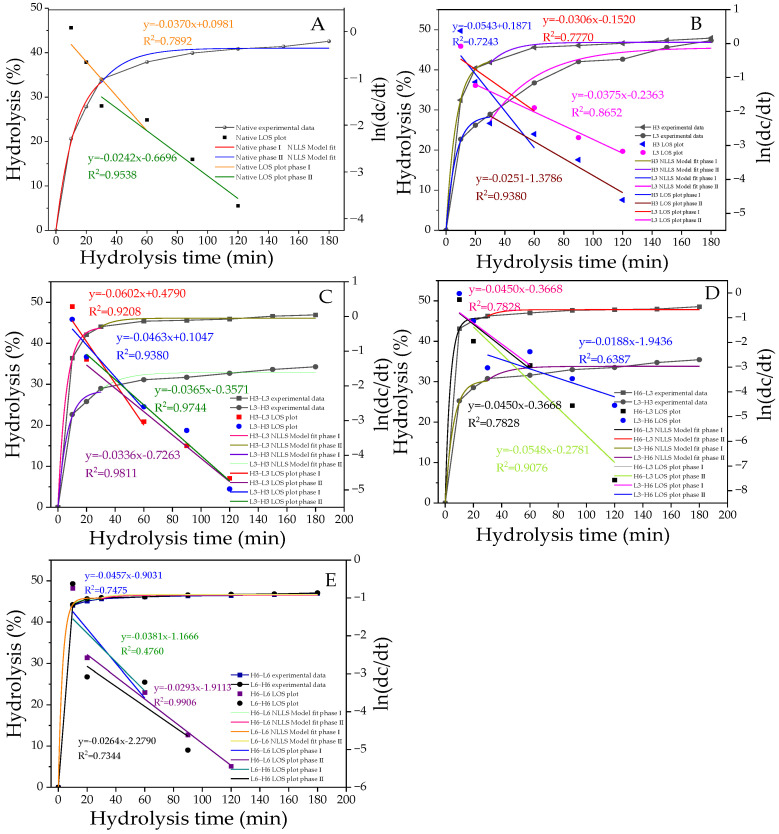
The digestion curves, model-fit curves, and LOS plots (**A**–**E**) of native and modified corn starch. All the LOS plots are divided into two stages, with straight lines of different slopes. The R^2^ values are related to the LOS plots.

In popular fitting models, the phase transition points of starch at different digestion stages can be obtained through LOS diagrams. Based on the fitting results, starch digestion can be divided into two parts: the fast digestion stage (stage I) and the slow digestion stage (stage II; Figure 4A–E), with the phase transition occurring at 30 min. The hydrolysis parameters (C_∞_ and K) obtained from the NLLS model are presented in Table 5. C_1∞_ and C_2∞_ represent the percentage of starch digested at the end of stages I and II, respectively. The K value, which is proportional to the reaction rate, indicates the speed of hydrolysis by enzymes during the digestive process [47]. As shown in Figure 4A–E, the hydrolysis rate of H-L starch was generally higher than that of L-H, confirming the results of the RDS, SDS, and RS (Figure 3). Similarly, for H/L6-L/H6 starches, the hydrolysis fitting curves largely overlapped, representing a relatively consistent degree of hydrolysis, which was associated with their highly similar SDS + RS content (Figure 3).

As shown in Table 5, the hydrolysis parameters of modified starch were significantly improved, in contrast with native corn starch. The L3 sample had a higher K_1_ and a lower K_2_ compared with the H3 sample. This was related to the higher RDS content and lower SDS content. The increase in RDS promoted the fast digestion stage, while the decrease in SDS reduced the speed of the slow digestion stage. The hydrolysis rate of the TD samples was significantly improved. Despite the expectation that TD samples with higher RC and ordered crystalline regions would be difficult to hydrolyze, the results showed the opposite. Higher K_1_ and K_2_ were found in the first-order kinetics. This was because the digestive enzyme only hydrolyzed amorphous and dispersed starch or weak crystallites and could not change the dense crystalline structure. Additionally, the pores and inward hilum formed by TD might elevate the digestion rate, resulting in higher K_1_ and K_2_, which was consistent with previous reports. This result emphasized the important effect of starch granule size and integrity on its digestibility [48].

### 3.10. Structure–Properties Relationship

The PCA technique was used to analyze the structure information, physicochemical characteristics, and in vitro digestion of corn starch. In Figure 3B, it is shown that the two principal components together explained 80.7% of the overall difference, with PC1 contributing 68.5% and PC2 contributing 12.2%. Native corn starch and modified starches did not overlap, suggesting significant differences between them. Native corn starch and H3 samples were located in the quadrant on the right side of PC1. Besides, TD samples, especially after heating for 6 h or freezing for 6 h, appeared in the groups on the left side of the PC1, while those from 3 h of heating and freezing were on the opposite side. This suggested that the duration of high-temperature treatment was an important factor.

Moreover, the A chain, RC, 1047/1022, and RS and, K_1_, K_2_, C_1∞_, and C_2∞_ showed clustering properties. The results indicated that the multi-scale structure had a close connection with the digestion properties and starch hydrolysis, and the relationship between A chain, RC, 1047/1022, and RS was confirmed in the aforementioned analyses (Section 3.4, Section 3.5, Section 3.7 and Section 3.8). Besides, the A chain, RC, and 1047/1022 were on the left side of PC2, showing a negative correlation with RDS. The loading diagram presented that the changes in digestive characteristics varied with changes in structure. Furthermore, the B_2_ chain was negatively correlated with SB, BD, TV, FV, PV, PT, and SDS, indicating that the structure had an impact on physicochemical and digestion properties. Therefore, the changes in physicochemical properties and digestion behavior depended on the structure information of corn starch.

### 3.11. Modification Mechanism

**Figure 5:**
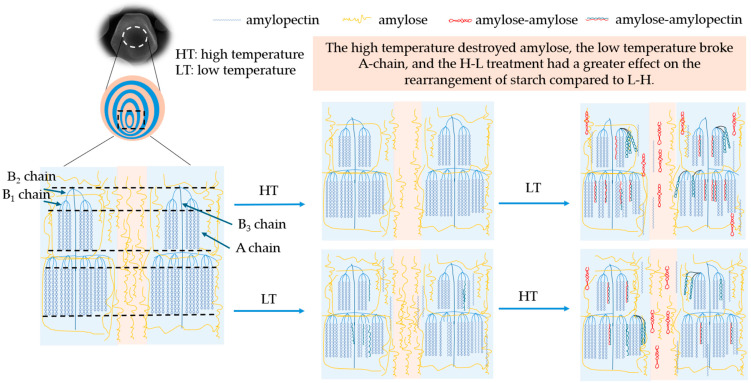
The mechanism diagram of native and modified corn starch.

The mechanism of the TD modification treatment time and temperature difference sequence on the multi-scale structure and physicochemical properties of corn starch is shown in Figure 5. Initially, starch granules consisted of growth rings alternating between crystalline and amorphous regions. After the H-L treatment, the fluorescence of the center of granules was dim, and the amylose in the umbilicus point of starch was damaged. The degradation of amylose was mainly caused by the thermal energy at 230 °C (Section 3.6). The short-chain content and crystallinity of starch treated with H-L increased, indicating the formation of more densely structured crystalline regions, which improved the anti-digestibility of corn starch (Section 3.3). With the prolongation of the temperature difference action time, the double-helix dissociation of amylopectin formed more short chains, which facilitated the interaction between amylose and amylopectin to form a denser crystalline structure. The mechanism was supported by increased crystallinity, short-range ordering, and resistant starch (Section 3.4, Section 3.5 and Section 3.8) [46].

In addition, the fluorescence of the starch umbilicus treated with L-H was more pronounced, indicating that there was an aggregation of amylose in the granule center. The freezing treatment at −80 °C destroyed some of the crystalline structure (Section 3.4) but retained a high content of amylose (Section 3.6). The high-temperature treatment in the later stage strengthened the connection between the crystalline and amorphous regions, making the overall structure more stable. Also, compared with H-L, L-H had more long chains in B_2_ and B_3_, and fewer short chains in A + B_1_, which limited the leaching of amylose at the gelatinization process and led to a decrease in viscosity. As the temperature treatment time prolonged, long chains significantly degraded to produce more short chains, leading to an increase in crystallinity and the formation of more ordered structures, thereby increasing the content of resistant starch (Section 3.3, Section 3.4, Section 3.5 and Section 3.8).

Therefore, H-L had a greater degree of rearrangement to the chain structure of starch compared with L-H starches. This result was proven by amylose content, amylopectin distribution, RC, and 1047/1022. In summary, the processing sequence of TD will affect the proportion of amylose/amylopectin and the difference in chain length distribution, leading to differences in starch crystal structure, short-range ordering, and viscosity characteristics. The processing time of TD affected the degradation degree of amylose and amylopectin, which resulted in a change in the physicochemical and digestive properties.

## 4. Conclusions

This study evaluated the effects of high temperature, low temperature, and their combination on the structure, physicochemical properties, and digestive behavior of corn starch. These results suggested that temperature difference modification influenced starch particles. TD enhanced the arrangement of the amorphous and crystalline regions, resulting in the clearness of the growth ring. During TD, a prolonged high temperature accelerated the starch umbilical concave inward, and an extended low temperature produced the micropores on the outer surface of starch particles. Furthermore, high temperature decreased the proportion of amylose, while low temperature reduced the A chain and disrupted the crystal structure of starch. TD increased the RC, short-range order, A and B_1_ chains, and hydrolysis parameters, and enhanced the stability of starch viscosity, converting SDS to RS and RDS. Overall, H-L had a stronger role of rearrangements on starch chains compared to L-H starch, and the processing time of TD showed a great effect on the degree of changes in amylose and amylopectin. The above results indicated that high temperature, low temperature, and TD could be used as an efficient physical process technology to increase the physicochemical characteristics and functions of starch.

## Figures and Tables

**Table 1:** Preparation information for all modified samples.

Samples’ Designation	Details of Sample Preparation
H3	The pretreated corn starch was heated at 230 °C for 3 h.
L3	The pretreated corn starch was subjected to −80 °C for 3 h.
H3-L3	The pretreated corn starch was heated at 230 °C for 3 h and then cooled at −80 °C for 3 h.
L3-H3	The pretreated corn starch was subjected to −80 °C for 3 h and then 230 °C for 3 h.
H6-L3	The pretreated corn starch was heated at 230 °C for 6 h and then cooled at −80 °C for 3 h.
L3-H6	The pretreated corn starch was modified at −80 °C for 3 h and then at 230 °C for 6 h.
H6-L6	The pretreated corn starch was heated at 230 °C for 6 h and then cooled at −80 °C for 6 h.
L6-H6	The pretreated corn starch was modified at −80 °C for 6 h and then at 230 °C for 6 h.

**Table 2:** Chain length distribution of the native and modified corn starch.

Samples	A (DP6-12)	B_1_ (DP13-24)	B_2_ (DP24-36)	B_3_ (DP ≥ 37)	A + B_1_
Native	12.55 ± 0.17 ^d^	42.31 ± 0.17 ^e^	19.53 ± 0.04 ^a^	25.61 ± 0.38 ^ab^	54.86 ± 0.34 ^ef^
H3	13.28 ± 0.64 ^d^	43.27 ± 0.16 ^de^	19.20 ± 0.26 ^abc^	24.25 ± 0.53 ^bc^	56.55 ± 0.79 ^de^
L3	11.19 ± 0.43 ^e^	42.66 ± 0.45 ^e^	19.59 ± 0.26 ^a^	26.54 ± 0.61 ^a^	53.86 ± 0.88 ^f^
H3-L3	13.25 ± 0.11 ^d^	43.80 ± 0.25 ^d^	19.40 ± 0.11 ^ab^	23.54 ± 0.24 ^c^	57.06 ± 0.35 ^d^
L3-H3	12.17 ± 0.27 ^de^	43.22 ± 0.14 ^de^	19.62 ± 0.07 ^a^	24.98 ± 0.35 ^abc^	55.39 ± 0.42 ^def^
H6-L3	17.26 ± 0.21 ^b^	49.57 ± 0.36 ^a^	17.78 ± 0.15 ^de^	15.38 ± 0.41 ^e^	66.84 ± 0.56 ^b^
L3-H6	15.25 ± 0.25 ^c^	47.73 ± 0.11 ^b^	18.55 ± 0.38 ^bcd^	18.47 ± 0.24 ^d^	62.98 ± 0.14 ^c^
H6-L6	18.90 ± 0.19 ^a^	50.04 ± 0.27 ^a^	17.28 ± 0.21 ^e^	13.78 ± 0.66 ^e^	68.94 ± 0.46 ^a^
L6-H6	16.66 ± 0.25 ^b^	46.43 ± 0.44 ^c^	18.50 ± 0.22 ^bcd^	18.40 ± 0.41 ^d^	63.09 ± 0.19 ^c^

Abbreviations: AC, amylose content. Values are means ± SD. Different letters within a column are significantly different (*p* < 0.05).

**Table 3:** The relative crystallinity, FTIR intensity ratio, and amylose content of the native and modified corn starch.

Samples	RC (%)	1047/1022	AC (%)
Native	25.46 ± 0.16 ^cd^	0.866 ± 0.002 ^bc^	23.03 ± 0.44 ^a^
H3	26.67 ± 0.38 ^bc^	0.879 ± 0.003 ^ab^	6.94 ± 0.95 ^d^
L3	22.49 ± 0.37 ^fg^	0.818 ± 0.009 ^d^	22.13 ± 0.43 ^a^
H3-L3	23.64 ± 0.30 ^ef^	0.874 ± 0.001 ^ab^	5.13 ± 0.14 ^de^
L3-H3	21.86 ± 0.13 ^g^	0.852 ± 0.005 ^c^	13.46 ± 1.37 ^b^
H6-L3	29.41 ± 0.82 ^a^	0.883 ± 0.004 ^ab^	3.89 ± 0.87 ^ef^
L3-H6	24.93 ± 0.23 ^de^	0.877 ± 0.001 ^ab^	9.13 ± 0.14 ^c^
H6-L6	30.46 ± 1.04 ^a^	0.891 ± 0.010 ^a^	2.65 ± 0.30 ^f^
L6-H6	27.52 ± 0.47 ^b^	0.885 ± 0.002 ^ab^	5.13 ± 0.89 ^de^

Values are means ± SD. Different letters within a line are significantly different (*p* < 0.05).

**Table 4:** The pasting parameters of native and modified corn starch.

Samples	PV (cp)	TV (cp)	BD (cp)	FV (cp)	SB (cp)	PT (min)	GT (°C)
Native	2811 ± 17 ^a^	2347 ± 26 ^a^	464 ± 9 ^a^	2864 ± 11 ^a^	517 ± 14 ^a^	5.40 ± 0.00 ^a^	77.63 ± 0.04 ^b^
H3	1061 ± 13 ^b^	873 ± 6 ^b^	188 ± 8 ^b^	996 ± 14 ^b^	123 ± 8 ^b^	5.34 ± 0.09 ^a^	84.78 ± 0.04 ^a^
L3	2836 ± 71 ^a^	2343 ± 2 ^a^	494 ± 69 ^a^	2868 ± 32 ^a^	525 ± 29 ^a^	5.34 ± 0.09 ^a^	77.50 ± 1.20 ^b^
H3-L3	1022 ± 2 ^b^	898 ± 4 ^b^	124 ± 1 ^b^	1019 ± 8 ^b^	121 ± 4 ^b^	5.40 ± 0.10 ^a^	85.70 ± 0.00 ^a^
L3-H3	821 ± 23 ^c^	652 ± 27 ^c^	169 ± 4 ^b^	769 ± 28 ^c^	116 ± 1 ^b^	5.34 ± 0.09 ^a^	84.38 ± 0.67 ^a^
H6-L3	46 ± 3 ^d^	27 ± 0 ^d^	19 ± 3 ^c^	41 ± 1 ^d^	14 ± 1 ^c^	4.17 ± 0.05 ^b^	/
L3-H6	32 ± 2 ^d^	15 ± 1 ^d^	17 ± 1 ^c^	22 ± 0 ^d^	7 ± 1 ^c^	3.80 ± 0.00 ^c^	/
H6-L6	46 ± 0 ^d^	29 ± 1 ^d^	18 ± 1 ^c^	43 ± 1 ^d^	14 ± 0 ^c^	4.20 ± 0.00 ^b^	/
L6-H6	24 ± 1 ^d^	10 ± 1 ^d^	15 ± 1 ^c^	17 ± 1 ^d^	7 ± 0 ^c^	3.83 ± 0.14 ^c^	/

Abbreviations: PV, peak viscosity; TV, trough viscosity; BD, breakdown; FV, final viscosity; SB, setback; PT, pasting time; GT, gelatinization temperature; /, not detected; K, digestion rate coefficient; C_∞_, percentage of starch digested at the end of the reaction (%). Values are means ± SD. Different letters within a line are significantly different (*p* < 0.05).

**Table 5:** The hydrolysis data of native and modified corn starch.

Samples	K_1_ (min^−1^)	C_1∞_ (%)	K_2_ (min^−1^)	C_2∞_ (%)
Native	0.08 ± 0.00 ^c^	37.02 ± 0.03 ^c^	0.05 ± 0.00 ^cd^	41.26 ± 0.07 ^d^
H3	0.14 ± 0.00 ^bc^	42.55 ± 0.02 ^b^	0.07 ± 0.00 ^bc^	46.85 ± 0.37 ^ab^
L3	0.15 ± 0.00 ^bc^	28.50 ± 0.46 ^d^	0.03 ± 0.00 ^d^	45.55 ± 0.65 ^c^
H3-L3	0.18 ± 0.02 ^bc^	43.58 ± 0.48 ^ab^	0.10 ± 0.00 ^ab^	46.11 ± 0.13 ^bc^
L3-H3	0.16 ± 0.05 ^bc^	28.48 ± 0.96 ^d^	0.07 ± 0.00 ^bc^	32.87 ± 0.24 ^e^
H6-L3	0.28 ± 0.03 ^ab^	45.42 ± 0.63 ^a^	0.12 ± 0.03 ^a^	47.49 ± 0.19 ^a^
L3-H6	0.17 ± 0.01 ^bc^	30.30 ± 0.80 ^d^	0.08 ± 0.00 ^bc^	33.80 ± 0.11 ^e^
H6-L6	0.35 ± 0.05 ^a^	45.40 ± 0.04 ^a^	0.13 ± 0.00 ^a^	46.50 ± 0.06 ^abc^
L6-H6	0.35 ± 0.09 ^a^	45.49 ± 0.05 ^a^	0.12 ± 0.01 ^a^	46.37 ± 0.05 ^abc^

Abbreviations: K, digestion rate coefficient; C_∞_, percentage of starch digested at the end of the reaction (%). Values are means ± SD. Different letters within a line are significantly different (*p* < 0.05).

## Data Availability

The original contributions presented in the study are included in the article, further inquiries can be directed to the corresponding author.

## References

[1] Ye S.J., Baik M.Y. (2023). Characteristics of physically modified starches. Food Sci. Biotechnol..

[2] Zhang J., Zhu X.F., Lu F., Yang Z., Tao H., Xu Y., Wang H.L. (2022). Physical modification of waxy maize starch: Combining SDS and freezing/thawing treatments to modify starch structure and functionality. Food Struct..

[3] Adedeji A.A., Ngadi M. (2018). Impact of freezing method, frying and storage on fat absorption kinetics and structural changes of parfried potato. J. Food Eng..

[4] Wang H.W., Xu K., Liu X.L., Zhang Y.Y., Xie X.H., Zhang H. (2021). Understanding the structural, pasting and digestion properties of starch isolated from frozen wheat dough. Food Hydrocoll..

[5] Tao H., Wang P., Ali B., Wu F., Jin Z., Xu X. (2015). Structural and functional properties of wheat starch affected by multiple freezing/thawing cycles. Starch/Stärke.

[6] Szymońska J., Krok F., Tomasik P. (2000). Deep-freezing of potato starch. Int. J. Biol. Macromol..

[7] Wang Y.C., Liang Y.C., Huang F.L., Chang W.C. (2023). Effect of Freeze-Thaw Cycles on Physicochemical and Functional Properties of Ginger Starch. Processes.

[8] Gong Y., Xu S., He T., Dong R., Hu X. (2020). Effect of quick-freezing temperature on starch retrogradation and ice crystals properties of steamed oat roll. J. Cereal Sci..

[9] Liu R., Yang Y.H., Cui X.J., Mwabulili F., Xie Y.L. (2023). Effects of Baking and Frying on the Protein Oxidation of Wheat Dough. Foods.

[10] Jain A., Passi S.J., Selvamurthy W., Singh A. (2019). Effect of frying temperature/frying cycles on trans-fats and oxidative stability of groundnut oil-cardiac risk factors. Asian J. Pharm. Clin. Res..

[11] Gong B., Xu M.J., Li B., Wu H., Liu Y., Zhang G.Q., Ouyang S.H., Li W.H. (2017). Repeated heat-moisture treatment exhibits superiorities in modification of structural, physicochemical and digestibility properties of red adzuki bean starch compared to continuous heat-moisture way. Food Res. Int..

[12] Zavareze E.D., Dias A.R.G. (2011). Impact of heat-moisture treatment and annealing in starches: A review. Carbohydr. Polym..

[13] Oktaviani S.R., Faridah D.N., Wulandari N., Afandi F.A., Jayanegara A. (2023). Resistant starch content of dual modification autoclaving-cooling and pullulanase debranching on various carbohydrate sources: A systematic review. Int. J. Food Sci. Technol..

[14] Almeida R.L.J., Santos N.C., Ferreira I.L.S., Pedro M.S., Feitoza J.V.F., Edu-ardo R.S., Freire V.A., Pereira T.S., de Sousa A.B.B., de Queiroga A.X.M. (2024). Effect of combined freezing with heat-moisture treatment (HMT) on the modification of in vitro digestibility, morphostructural, physicochemical, and thermal properties of Adzuki bean starch. J. Food Meas. Charact..

[15] Zhang H.F., Yin L., Wang Q.Y., Wang Y., Su J.M., Li C.M., Zhou X.Y. (2024). Effects of freeze–thaw cycles on the physicochemical and in vitro digestibility of starch in pre-fermented frozen raw dough. Int. J. Food. Sci. Technol..

[16] Xie D., Guo D., Guo Z., Hu X., Luo S., Liu C. (2021). Reduction of oil uptake of fried food by coatings: A review. Int. J. Food Sci. Technol..

[17] Daniel A.O., Matthews L., Reza T.A.O. (2020). Chicken processing by-product: A source of protein for fat uptake reduction in deep-fried chicken. Food Hydrocoll..

[18] Yang Q.Y., Lu X.X., Chen Y.Z., Luo Z.G., Xiao Z.G. (2019). Fine structure, crystalline and physicochemical properties of waxy corn starch treated by ultrasound irradiation. Ultrason. Sonochem..

[19] Zou J., Feng Y.T., Xu M.J., Yang P.Y., Zhao X.D., Yang B. (2023). The structure-glycemic index relationship of Chinese yam (*Dioscorea opposita* Thunb.) starch. Food Chem..

[20] Peng Y., Mao B., Zhang C., Shao Y., Wu T., Hu L., Hu Y., Tang L., Li Y., Tang W. (2021). Influence of physicochemical properties and starch fine structure on the eating quality of hybrid rice with similar apparent amylose content. Food Chem..

[21] Yao M., Tian Y., Yang W., Huang M., Zhou S., Liu X. (2019). The multi-scale structure, thermal and digestion properties of mung bean starch. Int. J. Biol. Macromol..

[22] He J.F., Li Y.J., Wang T., Deng Y.L., Wang S.B. (2023). Kinetic parameter estimation of hepatocellular carcinoma on 18 F-FDG PET/CT based on Bayesian method. Med. Phys..

[23] Shen H.S., Ge X.Z., Zhang B., Su C.Y., Zhang Q., Jiang H., Zhang G.Q., Yuan L., Yu X.Z., Li W.H. (2022). Preparing potato starch nanocrystals assisted by dielectric barrier discharge plasma and its multiscale structure, physicochemical and rheological properties. Food Chem..

[24] Zavareze E.D., Storck C.R., de Castro L.A.S., Schirmer M.A., Dias A.R.G. (2010). Effect of heat-moisture treatment on rice starch of varying amylose content. Food Chem..

[25] Zhang B., Saleh A.S.M., Su C., Gong B., Zhao K., Zhang G., Li W., Yan W. (2020). The molecular structure, morphology, and physicochemical property and digestibility of potato starch after repeated and continuous heat-moisture treatment. J Food. Sci..

[26] Zhao A., Yu L., Yang M., Wang C., Wang M., Bai X. (2018). Effects of the combination of freeze-thawing and enzymatic hydrolysis on the microstructure and physicochemical properties of porous corn starch. Food Hydrocoll..

[27] Liang W., Zhao W.Q., Liu X.Y., Zheng J.Y., Sun Z.Z., Ge X.Z., Shen H.S., Ospankulova G., Muratkhan M., Li W.H. (2022). Understanding how electron beam irradiation doses and frequencies modify the multiscale structure, physicochemical properties, and *in vitro* digestibility of potato starch. Food Res. Int..

[28] Chen P., Yu L., Simon G., Petinakis E., Dean K., Chen L. (2009). Morphologies and microstructures of corn starches with different amylose-amylopectin ratios studied by confocal laser scanning microscope. J. Cereal Sci..

[29] Hu A.J., Lu J., Zheng J., Sun J.Y., Yang L., Zhang X.Q., Zhang Y., Lin Q.Q. (2013). Ultrasonically aided enzymatical effects on the properties and structure of mung bean starch. Innov. Food Sci. Emerg..

[30] Sun X., Saleh A.S.M., Lu Y., Sun Z., Zhang X., Ge X., Shen H., Yu X., Li W. (2022). Effects of ultra-high pressure combined with cold plasma on structural, physicochemical, and digestive properties of proso millet starch. Int. J. Biol. Macromol..

[31] Shen H., Guo Y., Zhao J., Zhao J., Ge X., Zhang Q., Yan W. (2021). The multi-scale structure and physicochemical properties of mung bean starch modified by ultrasound combined with plasma treatment. Int. J. Biol. Macromol..

[32] Ge X., Shen H., Su C., Zhang B., Li W. (2021). The improving effects of cold plasma on multi-scale structure, physicochemical and digestive properties of dry heated red adzuki bean starch. Food Chem..

[33] Sievert D., Pomeranz Y. (1989). Enzyme-resistant starch I: Characterization and evaluation by enzymatic, thermoanalytical, and microscopic methods. Cereal Chem..

[34] Sun X., Sun Z., Guo Y., Yan W. (2021). Effect of twin-xuscrew extrusion combined with cold plasma on multi-scale structure, physicochemical properties, and digestibility of potato starches. Innov. Food Sci. Emerging Technol..

[35] Chen Y.Z., Yang Q.Y., Xu X.J., Qi L., Dong Z.H., Luo Z.G., Lu X.X., Peng X.C. (2017). Structural changes of waxy and normal maize starches modified by heat moisture treatment and their relationship with starch digestibility. Carbohydr. Polym..

[36] Wang H., Zhang B., Chen L., Li X. (2016). Understanding the structure and digestibility of heat-moisture treated starch. Int. J. Biol. Macromol..

[37] Gustavo1 L.V., Jose A.R., Consuelo L.C., Eduardo Jaime V.C., Nancy H.M. (2021). Characterization of Corn Starch-Calcium Alginate Xerogels by Microscopy, Thermal, XRD, and FTIR Analyses. Starch/Stärke.

[38] Zou J., Xu M.J., Zou Y.F., Yang B. (2020). Physicochemical properties and microstructure of Chinese yam (*Dioscorea opposita* Thunb.) flour. Food Hydrocoll..

[39] Ma Z., Boye J.I. (2018). Research advances on structural characterization of resistant starch and its structure-physiological function relationship: A review. Crit. Rev. Food Sci..

[40] Lemos P.V.F., Barbosa L.S., Ramos I.G., Coelho R.E., Druzian J.I. (2018). The important role of crystallinity and amylose ratio in thermal stability of starches. J. Therm. Anal. Calorims..

[41] Zhang G., Hamaker B.R. (2010). REVIEW: Cereal Carbohydrates and Colon Health. Cereal Chem..

[42] Rosell Cristina M., Yaiza B.-G. (2017). Comparison of porous starches obtained from different enzyme types and levels. Carbohydr. Polym..

[43] Englyst H.N., Kingman S.M., Cummings J.H. (1992). Classification and measurement of nutritionally important starch fractions. Eur. J. Clin. Nutr..

[44] Shin S.I., Lee C.J., Kim D.I., Lee H.A., Cheong J.J., Chung K.M., Baik M.Y., Park C.S., Kim C.H., Moon T.W. (2007). Formation, characterization, and glucose response in mice to rice starch with low digestibility produced by citric acid treatment. J. Cereal Sci..

[45] Lee C.J., Moon T.W. (2015). Structural characteristics of slowly digestible starch and resistant starch isolated from heat-moisture treated waxy potato starch. Carbohydr. Polym..

[46] Park E.Y., Baik B.K., Lim S.T. (2009). Influences of temperature-cycled storage on retrogradation and *in vitro* digestibility of waxy maize starch gel. J. Cereal Sci..

[47] Wang S.J., Wang S.K., Liu L., Wang S., Copeland L. (2017). Structural Orders of Wheat Starch Do Not Determine the *In Vitro* Enzymatic Digestibility. J. Agric. Food Chem..

[48] Dhital S., Shrestha A.K., Gidley M.J. (2010). Relationship between granule size and *in vitro* digestibility of maize and potato starches. Carbohydr. Polym..

